# Raising awareness and preparation for what may come: next of kin experiences of advance care planning with frail, home-dwelling older adults in geriatric units

**DOI:** 10.1186/s12913-025-12609-9

**Published:** 2025-03-27

**Authors:** Karin Berg Hermansen, Rigmor Einang Alnes, Trygve Johannes Lereim Saevareid, Reidar Pedersen, Siri Faerden Westbye, Maria Romøren, May Helen Midtbust

**Affiliations:** 1 Department of Health Sciences, Aalesund, Faculty of Medicine and Health Sciences, Norwegian University of Science and Technology, Box 1517, Aalesund, 6025 Norway; 2 Centre for Medical Ethics, Institute of Health and Society, University of Oslo, Box 1130 Blindern, Oslo, 0318 Norway

**Keywords:** Advance care planning, Older patients, Next of kin, Decision-making, Relational autonomy, Clinical ethics, Qualitative methods

## Abstract

**Background:**

Acutely ill and frail older adults and their next of kin are often poorly involved in planning of decisions regarding treatment and care during the final phase of life. Although advance care planning is a well-documented tool to strengthen patient autonomy and involve next of kin, it remains underused in hospital settings. We present a qualitative sub-study embedded in a cluster-randomized controlled trial, whose purpose was to implement advance care planning in Norwegian geriatric units. Frail, home dwelling older adults acutely admitted to geriatric hospital units were invited to participate in advance care planning together with their next of kin. The aim of this study was to explore next of kin experiences of advance care planning.

**Methods:**

The study has a qualitative design, based on individual semi-structured interviews with 13 next of kin. A purposive sampling was used to select next of kin who had recently participated in advance care planning from five geriatric units in the intervention arm. The analysis was conducted using reflexive thematic analysis by Braun and Clarke.

**Results:**

Four themes were developed from the analysis; (1) Being informed and involved through open communication; (2) Getting prepared for what’s to come; (3) The importance of the next of kin role in providing support and facilitation; (4) The need for documentation and collaboration across service levels.

**Conclusion:**

Advance care planning appears to provide a sense of security among next of kin by addressing their information needs regarding the patient’s prognosis, encouraging discussions on possible courses of action, and clarifying the patient’s end-of-life preferences. Next of kin played a crucial role in supporting the patient’s autonomy, and they considered the hospital stay as an ideal time for advance care planning. Increased awareness of their role as next of kin seems to enhance agreement and trust when confronting challenging situations and existential questions.

**Trial registration:**

ClinicalTrials.gov Identifier NTCT05681585. Registered 03.01.23.

**Supplementary Information:**

The online version contains supplementary material available at 10.1186/s12913-025-12609-9.

## Background

Advance care planning (ACP) is a voluntary communication process that enables adults to understand and share their personal values, life goals, and preferences regarding future health care before making decisions [1]. Through this process, ACP encourages respect for patients’ autonomy and their rights to be involved in decision-making [2]. Ideally, preferences and wishes should be discussed with healthcare personnel and next of kin, if possible [3]. Next of kin’s involvement is especially important in frail, older adults, where capacity to consent is reduced or likely to decline soon, due to multimorbidity [4] or dementia. The recent consensus of ACP regarding dementia emphasizes the importance of family’s involvement and suggests a flexible and pragmatic approach that allows collective decision making [5]. Next of kin may play a significant role in discussions regarding patients’ end-of-life care preferences to ensure they understand what is most important to the patient [6, 7]. The underlying goal of ACP is to strengthen relationships [8], and a relational view of autonomy recognizes the significance of relationships and the dynamic nature of autonomy in the context of chronic and severe illness [9].

In many countries, next of kin often make difficult end-of-life decisions for patients who are incapable of providing consent, and their own mental health is often affected in the process [10]. In Norway, the formal role of next of kin is limited to providing information about the preferences of patients who are incapable of making decisions, and treatment can only be administered if it is reasonably believed that the patient would have consented if capable and if the treatment aligns with the patient’s best interest. Thus, next of kin should be asked what the patients would have wanted before decision has to be made [11]. In practice, however, next of kin sometimes receive more decisive authority in medical decisions regarding life-prolonging treatment for incapacitated patients [12, 13], which may be perceived as a burden, particularly if the patient dies shortly after [14–16]. In addition, next of kin to frail older adults often serve as unpaid informal caregivers, thereby shouldering significant responsibilities. Their spouses, particularly, often shoulder the responsibility of providing care and support. Decisions regarding patients’ treatment and care can affect their well-being [17]. Meanwhile, next of kin need information and support from healthcare personnel to conduct their role. Therefore, involving next of kin through ACP can facilitate triadic communication and improve ACP and positive outcomes for the patient, next of kin, and healthcare personnel. For example, previous studies found that involvement in decision-making increased next of kin’s satisfaction and alleviated their caregiving burden [18] and they reported less stress and worry and felt better prepared for future responsibilities [19, 20]. Greater involvement of patients and their next of kin can help prevent conflicts, distrust, and over- and undertreatment [6, 21]. A survey of the general population in Norway with 1035 responses, revealed that 92% demonstrated a positive attitude towards ACP and expressed a desire for their next of kin to be involved [22].

International guidelines for ACP exist [3, 5, 23] and Norway has recently published its own national recommendations for ACP, and it emphasizes the importance of next of kin involvement [24]. Despite this, ACP remains underused in healthcare services and is poorly implemented in hospitals [25–27]. This may lead to insufficient involvement of acutely ill and frail older adults and their next of kin in treatment and care decisions during the final phase of life. A recent systematic review revealed that the experience of next of kin with ACP in frail older inpatients remains underexplored [25]. Gaining further insights is crucial for understanding the potential benefits of ACP for next of kin and how healthcare personnel can best utilize ACP to support both next of kin and patients. This study aimed to explore next of kin’s experiences with ACP in acute geriatric units.

## Methods

We followed the Standards for Reporting Qualitative Research (SRQR) in this article [28] (Additional file 1).

### Study design and context

This qualitative sub-study was conducted within a cluster-randomized controlled trial (CRCT): “Implementation of advance care planning in the routine care for acutely admitted patients in geriatric units” [29]. Twelve geriatric hospital units from the South-Eastern Norway Health Authority were randomly assigned to either the intervention or control arm. The intervention units assigned an implementation team (resource personnel), consisting of two to five healthcare personnel, receiving training and supervision, including practical exercises, from the research group. The project has a “whole ward” approach and resource personnel had responsibility to educate and train all healthcare personnel at their unit. Resources and toolkits such as ACP guidelines, pocket cards, information leaflets, documentation templates, and teaching materials were provided [29]. This sub-study provides in-depth data from next of kin’s perspective and focuses on the structured implementation of ACP. The conversations were led by a geriatrician, with nurses offering a more supportive role. In two of the ACP-conversations, another doctor was present to gain experience. Qualitative research during a trial can reveal the value of the intervention to important stakeholders [30] and explore participant benefits and possible disadvantages [31]. We used this method during the trial to explore next of kin’s experiences with ACP intervention. This study focused on the ACP- conversations that took place during admission, marking the patients’ very first ACP. In other sub- studies, we will report the experiences of the patients and clinicians *(forthcoming articles).*

### Individual interview- participants and data collection

Qualitative data were collected from five of the six geriatric units within the intervention arm. We used a purposive sampling strategy with criterion of inclusion [32], a natural choice for our study design, as we aimed to explore next of kins experiences of ACP in the intervention units. The inclusion criteria are shown in Table 1. Participants were recruited by the coordinator from the different geriatric units. These coordinators who were responsible for coordinating ACP activities at the unit, served as liaisons between the researchers and the unit, and were employed as nurses or geriatricians. In the invitation letter and orally, all participants were informed that their participation was voluntary and that they could withdraw at any time. Consent to participate was obtained from all participants.

**Table 1:** Inclusion criteria

**Next of kin**	**Older patient**
18 years or older Have participated in advance care planning in the hospital with the patient, physician, and preferably with a nurse. Next of kin of a patient who meets the inclusion criteria	70 years or older Home-dwelling Acutely admitted to the participated geriatric unit Clinical frailty scale score of 4 or higher* similar as the CRCT Capable of giving consent to participate in research The physician attending to the patients’ answers “no” to the “surprise question”: would you be surprised if the patient died within the first year? (from Gold Standards Framework proactive identification guidance) Conducted advance care planning in the hospital with next of kin, physician, and preferably also a nurse Not expected to die within 24 h Has not previously participated in ACP

During the inclusion period, 11–14 months after baseline, 13 next of kin were eligible for inclusion and were interviewed continuously. The individual interviews were conducted by first author KBH, and researcher SFW, with next of kin who had participated in ACP in geriatric units in the intervention arm. In one instance, two daughters of a patient attended and were interviewed together. The mean age was 64 years (46–92), and most were adult children of the patient (77%) (Table 2).

**Table 2:** Participants characteristics

**Study sample**	**Next of kin** *N* = 13	**%**
**Sex**		
Male	5	38.5
Female	8	61.5
**Age**		
45–55	5	38.5
56–65	3	23.0
66–75	3	23.0
76–95	2	15.5
**Relation**		
Spouse	3	23.0
Daughter	5	38.5
Son	5	38.5

The semi-structured interviews were conducted between September and December 2023, using the interview guide we developed (Additional file 2), inspired by previous research at the Centre for Medical Ethics [33]. Main question in Table 3 below.

**Table 3:** Main question in the interview guide

(i) What is your experience participating in the ACP-conversation? What aspects were positive or negative? (ii) What are your thoughts on the topic discussed? (iii) What significance do you think such a conversation can have? The guide also included questions about next of kin’s roles and areas of improvement.	

Most interviews were conducted within 8 days after ACP, with the latest occurring 48 days afterward. Each interview lasted for an average of 60 min and were conducted outside the hospital setting. Nine interviews were in person, six in private homes, and three in quiet public places; two were digitally with camera, and one was conducted over the phone at the participants’ request. For practical reasons and to increase recruitment, in six cases both next of kin and the patient were interviewed, but separately. We concluded the sub-study when the data held adequate information power. The interviews were audio-recorded, and the data-materials were immediately transferred to the University of Oslo’s Service for Sensitive Data (In Norwegian: “Tjenester for sensitive data,” TSD) for verbatim transcription.

### Analysis

To analyze data collected from the individual interviews we were inspired by the six step reflexive thematic methodology by Braun and Clarke [34, 35]. Field notes were written immediately after the interviews and incorporated into the analysis. All audio files were carefully listened to, and the transcripts were repeatedly read by the first author. Each interview was coded line by line, and single meanings were captured with supervision from MHM. NVivo 14 software [36] was used to code and organize the entire dataset. Further, the codes were organized and grouped together into initial themes, figured out shared pattern of meaning. These themes were reviewed and discussed collaboratively with REA, TJLS, and MHM. Throughout this phase, research questions guided the identification of meaningful themes, which were refined by iteratively revisiting the codes. The results were discussed in cooperation with all the authors.

### Reflexivity

As researchers involved in the main project (CRCT), the first author, along with TJLS, SFW, RP, and MR, contributed to implementation support. While familiarity with the project is advantageous, the themes of subjectivity and context were integral to the research process. During the interviews, we explored the disadvantages of ACP and sought suggestions for improvement. We present ourselves as “researchers” with no employment ties to the clinical team that conducted ACP.

## Results

Four themes were developed from and through coding in the analysis: (1) Being informed and involved through open communication; (2) Getting prepared for what’s to come; (3) The importance of the next of kin role in providing support and facilitation; (4) The need for documentation and collaboration across service levels.

### Being informed and involved through open communication

Next of kin emphasized the importance of being well-informed and involved in ACP alongside the patient. They valued receiving open and honest information about the patient’s condition, diagnosis, and treatment options. Several participants believed that transparency and clarity were crucial to good cooperation. A wife stated, “*We must be open and honest*,* so everything comes to light”* (ID9). The ACP theme included the patients’ ongoing medical treatment and health personnels’ perspectives on their prognosis. A son remarked, *“It’s about seeing possibilities or not possibilities in the future”* (ID1). They emphasized that this was a difficult discussion but *“something one has to endure”* (ID 9) and felt that it was positive and reassuring to be involved in the patient’s care trajectory.

*The whole process has been very*,* very positive. We got the information we needed and understood the situation. I thought it was very nice to be a part of it*,* even though it’s tough*,* but it’s just something one has to go through. It’s better to be involved than to be on the outside*,* not knowing… (Son*,* ID2).*

Some next of kin stated that they were surprised because their loved ones, queried by doctors, expressed that they did not want to receive information about their prognosis. One son said, *“If it was me*,* I would want to know”* (ID1), while another son expressed that being asked how much information one wanted was *“unbearable”* (ID4), as it intensified the uncertainty surrounding the disease’s progression.

Some next of kin noted the importance of timing in providing information. Some said that if ACP had occurred later, when the tests and MRI results were ready, due to the uncertainty of diagnosis, it would have been easier to discuss further processes. Nevertheless, they valued conversations and recognized the challenges of planning in a busy hospital unit.

Next of kin expressed satisfaction with the healthcare personnel. They emphasized the importance of equal communication during ACP and felt that the process was voluntary, with the patient controlling the topics discussed. Furthermore, the participants affirmed the healthcare personnel’s ability to respect and ensure the dignity of patients approaching the end of life through patient-centered discussions. All participants reported that healthcare personnel took time and prioritized conversations.

*They [healthcare personnel] were very skilled! Very. For me*,* it’s important that the elderly are respected and that they have a dignified end to life. I think that’s extremely important*,* and I believe there should be much more focus on it. Many elderly people are so overlooked… [alluding to previous experiences] (Daughter*,* ID12).*

Additionally, next of kin felt that their perspectives were valued and listened to. Both their loved ones and doctors actively engaged with them, seeking their opinions and input. They found that communication in ACP, the possibility of asking questions, and the empathetic listening of health personnel contributed to a sense of security and trust in the relationship between next of kin, patients, and healthcare personnel. Some highlighted that open communication and understanding can make life easier in challenging situations. One wife said: *“I think that the more one knows and the more one talks about it*,* the easier life becomes later*,* I believe*,* if things were to happen”* (ID7). Next of kin talked about the value of open communication and reassurance when facing difficult, challenging, and new situations at home. Some expressed concern about the patient’s condition and inability to cope with their care needs. A wife compared this to not being informed: *“So it is*,* you’re living on a kind of tightrope*,* one might almost say”* (ID8*).* After participating in ACP and gaining knowledge of her husband’s illness progression, she expressed:

*You become a little more reassured yourself*,* because… you’re at home worrying*,* right? I thought it was very nice. I think everyone should have such a conversation*,* so you don’t have to keep wondering*,* is it like this or is it like that*,* what’s going to happen now*,* and what are they planning to do… (Wife*,* ID8).*

Spouses with significant responsibilities highlighted that their voices were important to ACP. One wife expressed a desire for even greater participation in decision-making, particularly when it came to crucial choices, such as whether the patient should remain at home or consider respite stays or nursing home care. Still, spouses experienced that ACP also included *“how we both experienced it”* (ID7) and *“it was about our situation*” (ID9).

### Getting prepared for what’s to come

Next of kin appreciated that their loved ones’ hopes and worries played an important role in ACP. Through this discussion, they felt that ACP helped both the patients and their next of kin prepare for the last phase of life. One experienced ACP as an instruction manual for patient wishes and needs in the near future. ACP helped clarify what was important for the future and how they could contribute to the quality of life of their loved ones during the final phase of life.

*We’re in the final chapter*,* approaching the last stiff cover*,* and mother will be allowed to express what’s important in this final phase. It certainly raises awareness for me as next of kin… that we’re in this phase*,* actually… anything can happen here (Daughter*,* ID3).*

Next of kin described ACP as a process that raised awareness and preparation for what to come. One son stated that ACP prompted a more realistic understanding of his mother’s prognosis. Next of kin expressed that the open discussion in ACP with medical professionals helped them understand their loved one’s situation more fully. Some next of kin were not aware of the severity of the patient’s condition before ACP, and neither was the rest of the family. Through ACP, one son experienced that he was able to prepare the family for the reality that the patient’s condition was life-limiting. He expressed that this was an important preparation, allowing them to say their goodbyes before her passing.

Next of kin experienced that the patients’ reflections and wishes regarding the last phase of life and death were important themes in the conversation. A wife said that “*death and the end of life is a tough topic*,* like for everybody*,* but still important to talk about”* (ID9). A daughter described it as *“the big black difficult hole”* (ID3). A wife reported that patients’ thoughts about end of life were relevant questions in ACP, not because her husband “*should pass away the next day*” (ID9), but as an important preparation for both. Next of kin acknowledged that this phase was approaching but postponed discussing it. Next of kin emphasized that the timing when their loved ones were admitted to the hospital was relevant. One son said, “*It gives legitimacy to the conversation that it takes place in a hospital”* (ID4) *while another said: “It couldn’t have been more appropriate”* (ID2). They experienced hospital stay as a trigger for patients because it was a change in the situation, a deterioration, or a new diagnosis. One participant shared that his parent experienced fear of death on the first day of hospitalization. Next of kin perceived these factors as the reasons why patients were ready to discuss their situation, presenting a golden opportunity to perform ACP during their hospital stay.

Next of kin experienced that discussing death and mortality evoked a wide range of emotions and thoughts. Several next of kin mentioned that their previous experiences of grief in life made it easier to talk about death. Nevertheless, they emphasized that talking about existential feelings was challenging. However, they all highlighted and appreciated the fact that healthcare personnel facilitated dialogues that included patients’ thoughts about death and the last phase of life. Additionally, ensuring that this was conveyed in understandable language significantly contributed to their preparedness.

*For me*,* it was very crucial and reassuring that we approached a topic that*,* as a daughter*,* I find difficult to bring up. So*,* we kind of received some help in approaching something challenging*,* and I greatly appreciated that.… The doctor was very skilled at formulating*,* reformulating*,* and ensuring that the patient understood what was being asked (Daughter*,* ID3).*

Next of kin who had previous experiences with hospitalization and death in their immediate family expressed that ACP provided an opportunity for them to discuss these past experiences. Something they otherwise would not have talked about. Previous experiences, which are difficult and challenging, come into play in ACP. Some next of kin shared that their loved ones could openly discuss their fears about their final days and death. They experienced that these conversations were both relieving and valuable. Some next of kin had experienced overtreatment as medical interventions, and treatments as unnecessary stress for other family members at their end of life. Next of kin emphasized that they did not want treatment at any cost or futile treatment for their loved ones. Several participants mentioned that they wished they, retrospectively, had got the chance to communicate end-of-life issues earlier with those who have passed away. One son said that he wished that his late father had been given the opportunity to participate in ACP at the hospital earlier. He believed that if his father’s wishes were known, especially to healthcare personnel, it would have been easier to prevent overtreatment during the final phase of life. Next of kin emphasized the importance of healthcare personnel addressing significant dilemmas and asking important questions when individuals are near the end of life.

### The importance of the next of kin role in providing support and facilitation

Next of kin appreciated and were grateful for being invited to ACP in the geriatric unit. Some emphasized that health personnel should have expressed more clearly, when invited, how important it was for next of kin to participate given the significant existential themes discussed during ACP. Moreover, next of kin saw their role as important facilitators between patients and healthcare personnel in ACP. All next of kin emphasized that their loved ones should actively participate in decision-making. The primary role of next of kin was to listen to their loved ones’ preferences, values, and concerns during the conversation. Furthermore, they highlighted that their role was important in providing support and understanding to patients.

*I saw that it wasn’t easy for him [father]… I noticed that… but I believe it was good for him… that the most important thing was that he had us*,* the family*,* nearby*,* that we were there for him all the way (Son*,* ID2).*

Some had experienced that their loved ones wanted their opinions and support during ACP, especially when patients expressed wishes that they did not want more examinations or further medical treatments. However, being supportive could be challenging. They described these conversations as emotional, and one daughter said, *“I had to pull myself together”* (ID11). Participants broadly acknowledged that it was challenging for their loved ones to absorb all the information, particularly considering the patients prevalence of chronic diseases.

*My role was more to reassure her because she doesn’t have a very extensive vocabulary. So*,* she might feel a bit embarrassed in certain situations*,* especially when dealing with healthcare*,* as there are many terms and expressions that not everyone necessarily understands (Son*,* ID13)*.

Some noted that they were able to help the patient answer questions and offer assistance in engaging in conversations. One daughter shared that when her mother felt fatigued, she assisted her in communicating her priorities and preferences with healthcare personnel. This allowed her mother to express, confirm, or provide correct information.

Next of kin also emphasized the significance of their presence in helping patients remember crucial details discussed during ACP. They observed that their loved ones sometimes struggled to retell the conversation afterward, whereas next of kin retained information that their loved ones had not. Their involvement not only aids in remembering important details, but also facilitates follow-up. In addition, the ability to share this information with the rest of the family afterward was valuable. One daughter said, *“I feel fortunate to possess this knowledge*,* so I must share it with the other [siblings]”* (ID11).

Next of kin emphasized that they had gained a better awareness of their role after participating in ACP. One daughter said that participating in ACP was a positive experience for her. Through this process, she gained a deeper understanding of her mother’s independence, uncovered previously unknown wishes and thoughts, and acknowledged her own role in providing support.

### The need for documentation and collaboration across service levels

Next of kin talked about their further responsibility for fulfilling their loved ones wishes, both here and now, but also in the future: *“I feel that it is our responsibility now to continue helping*,* what Mom wants”* (ID12). They viewed documentation as a helpful aid for future decisions. During a home visit, the daughter found her mother’s written documentation of her ACP, which proved valuable. After her mother was discharged from the hospital, they continued to discuss any adjustments or changes that her mother might have wanted to make. Conversely, some next of kin missed the written summary of ACP if they did not receive it.

*It could have been a brief*,* written summary… some keywords*,* what were the main points*,* a journal note. And if there were to be a recurring conversation*,* that conversation could have started with these points. What the main conclusion is*,* are you in agreement with those*,* is that how we understood you? (Son*,* ID5).*

Next of kin emphasized the importance of information sharing in ACP and collaboration among healthcare professionals at different levels, including the specialist health service and municipal health and care services. Documentation was mentioned as crucial, given their responsibility, “*to be the bearer of what is my mother desire*” (ID3), to convey their loved one’s wishes. They emphasized the importance of ensuring that others are aware of their loved ones’ preferences and can assist in making decisions, if necessary. Next of kin expressed uncertainty regarding their roles in decision making if their loved ones were to lose the capacity to participate in the future. However, advocating for loved ones was seen as important. Some would *“raise my voice for my father*,* if I had felt that he is not being heard”* (ID6). Moreover, next of kin expressed that they would consider different perspectives but ultimately *“take into account what our mother would have wanted and make the best decision based on that”* (ID12). Next of kin expressed that the documentation of ACP was seen as a security. One daughter said the following about her role if her mother had lost decision-making capacity.

*With this conversation*,* I have a framework that I can provide to healthcare professionals. Having it documented in the summary feels secure*,* and if this situation arises*,* I can confidently say that we had an ACP conversation*,* referring to its existence*,* and emphasize that it’s my mother’s decision (Daughter*,* ID3).*

Next of kin believed that having documented information would make it easier to assist patients in fulfilling their wishes in the future. As one daughter said it, *“the documentation provides more power in challenging situations”* (ID3). The participants highlighted that increased responsibility could lead to ethical challenges in the context of future decision-making. Some discussed difficulties arising from different opinions among siblings or family members regarding what was best for their loved ones. One daughter shared that she and her parents held contrasting views.

*My father didn’t want to be treated for it [cancer]. You felt*,* in a way*,* that there was hope*,* that they could figure things out. But then my father slowed down; he didn’t want any further investigations*,* no more treatment except pain relief.*

*At the same time*,* from my perspective*,* I have to see it from his point of view. He wants to avoid many days of pain*,* and for it to be over quickly. While I want to have him in my life (Daughter*,* ID6).*

Balancing different perspectives and emotions while making difficult decisions has been described as challenging. A wife said that ACP had *“set emotions in motion*,* but there was no follow-up”* (ID9). The patient’s need for emotional processing was also mentioned by a daughter:

*It could have been good to follow up with a couple of conversations afterward. At least when you talk about it so consciously*,* I think you need to process it afterward (Daughter*,* ID10).*

According to the participant, she had been invited without getting a brochure on ACP content, nor had she received oral information. Still, she found ACP to be valuable but wished that they had been more prepared for the themes and purpose of ACP.

Some next of kin received information that ACP could be repeated, but they were unsure about who would be involved. A son described his loved one’s condition as “*a life situation in which there are still many choices*” (ID1). Nevertheless, most next of kin expressed no desire or need for a new conversation but would be grateful to participate if their loved one’s situation changed.

## Discussion

In this study, we explored next of kin’s experiences of participating in an ACP-conversation in acute geriatric units. The key findings include that ACP seems to address the need for next of kin to be informed about the patient’s prognosis, facilitate discussions about potential healthcare and treatment options for the present and future, and clarify the patient’s end-of-life preferences. Next of kin experienced that participating in ACP not only involved them actively but also allowed them to play a further, crucial role in ensuring that their loved ones’ wishes were respected. Furthermore, they felt increased responsibility for honoring their loved ones’ preferences and saw documentation and cooperation as crucial.

### Informed and involved in a triad

Next of kin emphasized the importance of being well-informed and actively involved in ACP, together with the patient and healthcare personnel. Within this triad, we found that next of kin were helped by healthcare personnel in broaching sensitive topics. Next of kin emphasized the significance of healthcare personnel asking patients open-ended questions about their values, views on treatment, end-of-life considerations, and death. Discussing patients’ existential concerns can be challenging and can be a barrier to ACP and patient autonomy [36, 37]. The importance of healthcare personnel in initiating ACP is consistent with earlier research [38]. This study shows the value of a patient-centered approach, exemplified by raising existential concerns and allowing the patient to express their desires through active listening, and this is also compatible with including next of kin in ACP. This has increased awareness among next of kin about factors that may be important to patients during their final phase of life.

The study revealed that ACP empowered next of kin to express empathy toward their loved ones because the patients wanted their support. Strengthening relations through ACP, where patients wanted support from next of kin, appeared to foster greater consensus and better prepare next of kin for their role. This approach aligns with the findings of a previous study, emphasizing the importance of a relational autonomy perspective [9]. This underscores the interconnectedness, vulnerability, and mutual reliance on support. An individual’s autonomy is shaped and influenced by our interactions with others. Especially in end-of-life practices, patients are situated in the center, interacting with both healthcare personnel and family, and a patient-doctor-family triad is recommended [39]. Additionally, encouraging open discussions among families about their preferences is recommended to improve decision-making processes [40, 41]. The significant role of next of kin in our study was demonstrated through respect for the older patient’s autonomy, assistance in participation, and support and respect for the patient’s wishes. In this study, it appears that gaining a deeper understanding of loved ones’ autonomy, rights, health situations, possible courses of action, and preferences led to a more supportive role and improved triadic collaboration.

### Preparation and future decision-making

Next of kin talked about ACP as raising awareness and preparing for what may come. ACP provide emotional support to next of kin. This support may enable next of kin and their loved ones to proactively prepare for the future and empower patients to make well-informed decisions regarding their care. Our findings revealed that ACP fulfilled an unmet need for next of kin by providing a platform for information exchange and involvement in their loved one’s illness trajectory. Some next of kin were surprised by the patient’s frailty and poor prognosis, while this was well-known to healthcare personnel. When a patient’s condition is at high risk of deterioration, the responsibility for their next of kin may increase. Next of kin in our study were not well acquainted with their role in decision-making if the patient is incapacitated. Findings from a Norwegian study found that 78% of next of kin thought they were responsible for medical treatment decisions [42]. If patients become incapacitated, the next of kin may bear the burden of being their voice without knowing their preferences or having received adequate information from healthcare personnel. After ACP, next of kin in this study reported an increased understanding of their loved ones’ preferences and a clearer sense of their own responsibilities.

ACP may not consistently align with future scenarios that individuals encounter when it comes to specific treatment preferences. Achieving concordance between patient wishes and the treatment provided at the end of life can be challenging [43]. However, other outcomes may be important. Consequently, there is an ongoing international discussion on redefining goals in ACP [44], emphasizing the value of ACP as a relational and communicative process [45]. In 2010, Sudore et al. suggested to change the “p” in ACP from planning to preparation [46], a position supported by Malhotra and Heyland [47, 48]. A caregiver-focused approach, which helps family “understand how they can help to shape the final chapter of a patient’s life story” [49], and reduce surrogate (i.e. family) distress [43] is considered more appropriate. Focusing on the actual process, addressing hopes and worries and emphasizing that expressing oneself and being cared for are paramount [47]. Still, use illness trajectories to include patient input and values, and priorities are discussed in case of sudden changes [50]. In this study, it appeared that patients’ specific wishes were crucial. Additionally, discussions, including those about patients’ short-term wishes within ACP, can serve as a valuable starting point for next of kin to understand patients’ attitudes and values, potentially providing a better basis for current and future decision-making.

Communication and understanding within families can be complex, especially during difficult times. Different views and uncertainties about treatment options can hinder patient autonomy and foster ethical challenges [51, 52] and family concerns may be given greater weight than those of patients [53]. This study does not provide answer to the long-term effects, while next of kin were interviewed shortly after the ACP-conversation. However, next of kin in this study found that ACP provided space for discussing important issues ahead of time. Some patients expressed a preference for pain relief and discontinuation of medical treatments, such as chemotherapy, rather than prioritizing life extension. This study indicates that, in ACP, within this triad, next of kin felt acknowledged and heard. Open communication regarding patients’ illness trajectories, treatment options, and prognoses helped prepare next of kin and potentially bridged the gap between their expectations and those of their loved ones. Discussing these issues in advance can alleviate stress for next of kin in acute settings or prior to the patient losing capacity or dying and has been shown to improve trust and understanding [54, 55].

Documenting ACP is crucial for ensuring collaboration on end-of-life decisions across various services. It makes the frail older patient’s wishes accessible to clinicians and other family-members who were not present during the ACP conversation. Furthermore, it allows these wishes to be respected in the future, even if the older adult can no longer participate in decision-making. The documentation also makes it easier for next of kin present during ACP to share important information with others, enabling continuity in future ACP. Additionally, it may provide reassurance to all stakeholders and help prevent conflicts, including among relatives [56]. Recognizing the inevitability of impending death can facilitate crucial conversations, provide closure, and foster acceptance.

### Timing: a golden opportunity

Being prepared is possible if healthcare personnel involve next of kin early on. However, the timing of ACP poses challenges owing to frailty and unpredictable illness trajectories in older adults. Conversations regarding end-of-life matters tend to occur late in patients’ disease trajectories [57]. Our study revealed that next of kin preferred geriatric units as the ideal setting for these discussions. Interestingly, next-of-kin experienced that emergency hospital admissions often acted as triggers for loved ones to engage in ACP, consistent with previous research [58, 59]. Notably, initiating these conversations in nursing homes may be too late to include the patient’s voice directly because of the high incidence of cognitive impairment [60]. Implementing ACP in geriatric units and involving next of kin according to patients’ own wishes may mitigate missed opportunities and support and increase the patient’s ability to participate in ACP. This study revealed that next of kin have an important role, both in ACP but also further responsibility to be their loved one’s voice when needed. The preparation, awareness, and understanding of their loved ones’ hopes and values seem to be vital for next of kin. Furthermore, next of kin may play a key role in ACP or similar conversations when the patient is unable to participate.

### Strengths and limitations

Exploring an individual’s next of kin’s subjective and situation-specific knowledge of ACP participation may contribute to practice validity. Although this result is specific to a small group of selected participants, the knowledge can be useful for healthcare personnel conducting ACP. The main strengths of this sub-study are its unique position alongside a CRCT and the fact that the results originate from a context in which ACP is new. The results of our study should be interpreted in light of several limitations. The participants experiences may not reflect the real word outside the intervention units, and the participants may have been more positive than average. We have explored next of kins experiences shortly after one ACP-conversation, but we have not explored the long-term effects of ACP.

## Conclusions

Advance care planning provides next of kin with a sense of security by addressing their informational needs regarding the patient’s prognosis and facilitating discussions on potential actions and end-of-life preferences. This study highlights the importance of relationships in decision-making dynamics, with next of kin playing a crucial role in supporting patient autonomy. They emphasize the need for healthcare personnel to initiate conversations about existential concerns. A triadic approach to ACP can better prepare next of kin and initiate the necessary processes before decisions become urgent. Establishing a shared understanding between next of kin and healthcare personnel may prevent unnecessary interventions that can compromise patients’ quality of life and autonomy. The study results indicate that healthcare personnel should invite and involve next of kin in ACP for frail, home-dwelling older inpatients if the patients consent. It will be essential also to explore the perspectives of frail older patients and healthcare personnel with this triadic approach to ACP in geriatric units. Further research on benefits and disadvantages, and on documentation of ACP and collaboration between different levels is also needed.

## Supplementary Information

Additional file 1. Standards for Reporting Qualitative Research (SRQR).

Additional file 2. Interview guide.

## Data Availability

The dataset will be de-identified or deleted before the end of the study period, in line with the research ethics approval and consent. If it is possible to de-identify, and subsequently anonymize the data, they will be made publicly available.

## Abbreviations

ACP: Advance care planning
CRCT: Cluster randomized controlled trial
REC: Regional committee for Medical and Health Research Ethics
SIKT: Norwegian Agency for Shared Services in Education and Research
TSD: Tjenester for Sensitive Data

## References

[1] Sudore RL, Lum HD, You JJ, Hanson LC, Meier DE, Pantilat SZ, et al. Defining advance care planning for adults: A consensus definition from a multidisciplinary Delphi panel. J Pain Symptom Manage. 2017;53(5):821–e321. 10.1016/j.jpainsymman.2016.12.331. 28062339 10.1016/j.jpainsymman.2016.12.331 PMC5728651

[2] Beauchamp T, Childress J. Principles of biomedical ethics: marking its fortieth anniversary. Am J Bioeth. 2019;19(11):9–12. 10.1080/15265161.2019.1665402. 31647760 10.1080/15265161.2019.1665402

[3] Rietjens JAC, Sudore RL, Connolly M, van Delden JJ, Drickamer MA, Droger M, et al. Definition and recommendations for advance care planning: an international consensus supported by the European association for palliative care. Lancet Oncol. 2017;18(9):e543–51. 10.1016/s1470-2045(17)30582-x. 28884703 10.1016/S1470-2045(17)30582-X

[4] Wang X, Huang XL, Wang WJ, Liao L. Advance care planning for frail elderly: are we missing a golden opportunity? A mixed-method systematic review and meta-analysis. BMJ Open. 2023;13(5):e068130. 10.1136/bmjopen-2022-068130. 37247960 10.1136/bmjopen-2022-068130 PMC10230943

[5] van der Steen JT, Nakanishi M, Van den Block L, Di Giulio P, Gonella S, In der Schmitten J, et al. Consensus definition of advance care planning in dementia: A 33-country Delphi study. Alzheimers Dement. 2023. 10.1002/alz.13526. 37985444 10.1002/alz.13526 PMC10916978

[6] Malhotra C, Shafiq M, Batcagan-Abueg APM. What is the evidence for efficacy of advance care planning in improving patient outcomes? A systematic review of randomised controlled trials. BMJ Open. 2022;12(7):e060201. 10.1136/bmjopen-2021-060201.

[7] Houben CHM, Spruit MA, Groenen MTJ, Wouters EFM, Janssen DJA. Efficacy of advance care planning: A systematic review and Meta-Analysis. J Am Med Dir Assoc. 2014;15(7):477–89. 10.1016/j.jamda.2014.01.008. 24598477 10.1016/j.jamda.2014.01.008

[8] Fleuren N, Depla MFIA, Janssen DJA, Huisman M, Hertogh CMPM. Underlying goals of advance care planning (ACP): a qualitative analysis of the literature. BMC Palliat Care. 2020;19(1):27. 10.1186/s12904-020-0535-1. 32143601 10.1186/s12904-020-0535-1 PMC7059342

[9] Killackey T, Peter E, Maciver J, Mohammed S. Advance care planning with chronically ill patients: A relational autonomy approach. Nurs Ethics. 2020;27(2):360–71. 10.1177/0969733019848031. 31122121 10.1177/0969733019848031

[10] Malhotra C, Huynh VA, Shafiq M, Batcagan-Abueg APM. Advance care planning and caregiver outcomes: intervention efficacy - systematic review. BMJ Support Palliat Care. 2022. 10.1136/spcare-2021-003488. 10.1136/spcare-2021-003488 35788465

[11] omsorgsdepartementet H-o. Patient Rights Act. 1999. https://lovdata.no/lov/1999-07-02-63. Accessed Aug 2024.

[12] Romøren M, Pedersen R, Førde R. How do nursing home Doctors involve patients and next of kin in end-of-life decisions? A qualitative study from Norway. BMC Med Ethics. 2016;17(1):5. 10.1186/s12910-016-0088-2. 26769024 10.1186/s12910-016-0088-2 PMC4714479

[13] Dreyer A, Forde R, Nortvedt P. Autonomy at the end of life: life-prolonging treatment in nursing homes—relatives’ role in the decision-making process. J Med Ethics. 2009;35(11):672–7. 10.1136/jme.2009.030668. 19880703 10.1136/jme.2009.030668

[14] Midtbust MH, Alnes RE, Gjengedal E, Lykkeslet E. Separation characterized by responsibility and guilt: family caregivers’ experiences with palliative care for a close family member with severe dementia in long-term care facilities. Dementia. 2021;20(2):518–33. 10.1177/1471301219898341. 31918569 10.1177/1471301219898341

[15] Fosse A, Schaufel MA, Ruths S, Malterud K. End-of-life expectations and experiences among nursing home patients and their relatives—A synthesis of qualitative studies. Patient Educ Couns. 2014;97(1):3–9. 10.1016/j.pec.2014.05.025. 24976628 10.1016/j.pec.2014.05.025

[16] Thoresen L, Pedersen R, Lillemoen L, Gjerberg E, Førde R. Advance care planning in Norwegian nursing homes-limited awareness of the residents’ preferences and values? A qualitative study. BMC Geriatr. 2019;19(1):363. 10.1186/s12877-019-1378-6. 31870302 10.1186/s12877-019-1378-6 PMC6929496

[17] Carr D, Moorman SM, Boerner K. End-of-life planning in a family context: does relationship quality affect whether (and with whom) older adults plan? J Gerontol B Psychol Sci Soc Sci. 2013;68(4):586–92. 10.1093/geronb/gbt034. 23689997 10.1093/geronb/gbt034 PMC3674735

[18] Ris I, Schnepp W, Mahrer Imhof R. An integrative review on family caregivers’ involvement in care of home-dwelling elderly. Health Soc Care Community. 2019;27(3):e95–111. 10.1111/hsc.12663. 30307685 10.1111/hsc.12663

[19] Detering KM, Hancock AD, Reade MC, Silvester W. The impact of advance care planning on end of life care in elderly patients: randomised controlled trial. BMJ. 2010;340(7751). 10.1136/bmj.c1345. 10.1136/bmj.c1345 PMC2844949 20332506

[20] Jimenez G, Tan WS, Virk AK, Low CK, Car J, Ho AHY. Overview of systematic reviews of advance care planning: summary of evidence and global lessons. J Pain Symptom Manage. 2018;56(3):436–e5925. 10.1016/j.jpainsymman.2018.05.016. 29807158 10.1016/j.jpainsymman.2018.05.016

[21] Brinkman-Stoppelenburg A, Rietjens JAC, van der Heide A. The effects of advance care planning on end-of-life care: A systematic review. Palliat Med. 2014;28(8):1000–25. 10.1177/0269216314526272. 24651708 10.1177/0269216314526272

[22] Sævareid TJL, Pedersen R, Magelssen M. Positive attitudes to advance care planning - a Norwegian general population survey. BMC Health Serv Res. 2021;21(1):762. 10.1186/s12913-021-06773-x. 34334131 10.1186/s12913-021-06773-x PMC8327435

[23] The Gold Standards Framework. Advance care planning. https://www.goldstandardsframework.org.uk/advance-care-planning. Accessed Oct 2023.

[24] Helsedirektoratet. Forhåndssamtaler og planlegging ved begrenset forventet levetid. 2023. https://www.helsedirektoratet.no/faglige-rad/Forhandssamtaler-og-planlegging-ved-begrenset-forventet-levetid.

[25] Hopkins SA, Bentley A, Phillips V, Barclay S. Advance care plans and hospitalized frail older adults: a systematic review. BMJ Supportive Palliat Care. 2020;10(2):164. 10.1136/bmjspcare-2019-002093. 10.1136/bmjspcare-2019-002093 PMC7286036 32241957

[26] Block BL, Jeon SY, Sudore RL, Matthay MA, Boscardin WJ, Smith AK. Patterns and trends in advance care planning among older adults who received intensive care at the end of life. JAMA Intern Med. 2020;180(5):786–9. 10.1001/jamainternmed.2019.7535. 32119031 10.1001/jamainternmed.2019.7535 PMC7052782

[27] Sævareid TJL, Aasmul I, Hjorth NE. Implementation of advance care planning in Norway. Zeitschrift Für Evidenz Fortbildung Und Qualität Im Gesundheitswesen. 2023. 10.1016/j.zefq.2023.05.017. 10.1016/j.zefq.2023.05.017 37394337

[28] O’Brien BC, Harris IB, Beckman TJ, Reed DA, Cook DA. Standards for reporting qualitative research: A synthesis of recommendations. Acad Med. 2014;89(9):1245–51. 10.1097/acm.0000000000000388. 24979285 10.1097/ACM.0000000000000388

[29] Romøren M, Hermansen KB, Sævareid TJL, Brøderud L, Westbye SF, Wahl AK, et al. Implementation of advance care planning in the routine care for acutely admitted patients in geriatric units: protocol for a cluster randomized controlled trial. BMC Health Serv Res. 2024;24(1):220. 10.1186/s12913-024-10666-0. 38374100 10.1186/s12913-024-10666-0 PMC10875743

[30] O’Cathain A, Thomas KJ, Drabble SJ, Rudolph A, Hewison J. What can qualitative research do for randomised controlled trials? A systematic mapping review. BMJ Open. 2013;3(6). 10.1136/bmjopen-2013-002889. 10.1136/bmjopen-2013-002889 PMC3669723 23794542

[31] Lewin S, Glenton C, Oxman AD. Use of qualitative methods alongside randomised controlled trials of complex healthcare interventions: methodological study. BMJ. 2009;339:b3496. 10.1136/bmj.b3496. 19744976 10.1136/bmj.b3496 PMC2741564

[32] Palinkas LA, Horwitz SM, Green CA, Wisdom JP, Duan N, Hoagwood K. Purposeful sampling for qualitative data collection and analysis in mixed method implementation research. Adm Policy Ment Health. 2015;42(5):533–44. 10.1007/s10488-013-0528-y. 24193818 10.1007/s10488-013-0528-y PMC4012002

[33] Hestmark L, Romøren M, Heiervang KS, Weimand B, Ruud T, Norvoll R, et al. Implementation of guidelines on family involvement for persons with psychotic disorders in community mental health centres (IFIP): protocol for a cluster randomised controlled trial. BMC Health Serv Res. 2020;20(1):934. 10.1186/s12913-020-05792-4. 33036605 10.1186/s12913-020-05792-4 PMC7547488

[34] Braun V, Clarke V. Using thematic analysis in psychology. Qualitative Res Psychol. 2006;3:77–101. 10.1191/1478088706qp063oa.

[35] Braun V, Clarke V. Conceptual and design thinking for thematic analysis. Qualitative Psychol (Washington DC). 2021;9(1):3–26. 10.1037/qup0000196.

[36] Lumivero. NVivo (Version 14). 2023. https://lumivero.com/.

[37] Agledahl KM, Gulbrandsen P, Førde R, Wifstad Å. Courteous but not curious: how Doctors’ politeness masks their existential neglect. A qualitative study of video-recorded patient consultations. J Med Ethics. 2011;37(11):650–4. 10.1136/jme.2010.041988. 21610269 10.1136/jme.2010.041988 PMC3198010

[38] Vandenbogaerde I, Cohen J, Hudson P, Van Audenhove C, Deliens L, De Vleminck A. Support from healthcare professionals in empowering family carers to discuss advance care planning: A population-based survey. Palliat Med. 2023;37(5):719–29. 10.1177/02692163221135032. 36349646 10.1177/02692163221135032

[39] Gómez-Vírseda C, de Maeseneer Y, Gastmans C. Relational autonomy in end-of-life care ethics: a contextualized approach to real-life complexities. BMC Med Ethics. 2020;21(1):50. 10.1186/s12910-020-00495-1. 32605569 10.1186/s12910-020-00495-1 PMC7325052

[40] Combes S, Gillett K, Norton C, Nicholson CJ. The importance of living well now and relationships: A qualitative study of the barriers and enablers to engaging frail elders with advance care planning. Palliat Med. 2021;35(6):1137–47. 10.1177/02692163211013260. 33934669 10.1177/02692163211013260 PMC8189003

[41] Le TD, Lin S-C, Huang M-C, Fan S-Y, Kao C-Y. Factors impacting the demonstration of relational autonomy in medical decision-making: A meta-synthesis. Nurs Ethics. 2023;09697330231200570. 10.1177/09697330231200570. 10.1177/09697330231200570 37818823

[42] Aarseth S, Horn MA, Dahlberg J, Morten M. The population’s knowledge of and attitudes towards end-of-life decisions. 2023. https://www.michaeljournal.no/asset/issue/2023/01/Michael-2023-01_011-023-eng.pdf.

[43] McMahan RD, Tellez I, Sudore RL. Deconstructing the complexities of advance care planning outcomes: what do we know and where do we go?? A scoping review. J Am Geriatr Soc. 2021;69(1):234–44. 10.1111/jgs.16801. 32894787 10.1111/jgs.16801 PMC7856112

[44] Periyakoil VS, Gunten CFv, Arnold R, Hickman S, Morrison S, Sudore R. Caught in a loop with advance care planning and advance directives: how to move forward?? J Palliat Med. 2022;25(3):355–60. 10.1089/jpm.2022.0016. 35230896 10.1089/jpm.2022.0016 PMC9022450

[45] Pollock K, Bulli F, Caswell G, Kodba-Čeh H, Lunder U, Miccinesi G, et al. Patient and family caregiver perspectives of advance care planning: qualitative findings from the ACTION cluster randomised controlled trial of an adapted respecting choices intervention. Mortality. 2022;1–21. 10.1080/13576275.2022.2107424.

[46] Sudore RL, Fried TR. Redefining the planning in advance care planning: Preparing for end-of-life decision making. Ann Intern Med. 2010;153(4):256–61. 10.7326/0003-4819-153-4-201008170-00008. 20713793 10.1059/0003-4819-153-4-201008170-00008 PMC2935810

[47] Malhotra C. Advance care planning: it is time to rethink our goals. J Am Geriatr Soc. 2023. 10.1111/jgs.18511. 37522615 10.1111/jgs.18511

[48] Heyland DK. Advance care planning (ACP) vs. Advance serious illness preparations and planning (ASIPP). Healthcare. 2020;8(3):218. 10.3390/healthcare8030218. 32708449 10.3390/healthcare8030218 PMC7551637

[49] Fried TR. Giving up on the objective of providing goal-concordant care: advance care planning for improving caregiver outcomes. J Am Geriatr Soc. 2022;70(10):3006–11. 10.1111/jgs.18000. 35974460 10.1111/jgs.18000 PMC9588724

[50] Murray SA, Boyd K, Moine S, Kendall M, Macpherson S, Mitchell G, et al. Using illness trajectories to inform person centred, advance care planning. BMJ. 2024;384:e067896. 10.1136/bmj-2021-067896. 38428953 10.1136/bmj-2021-067896

[51] Mullick A, Martin J, Sallnow L. An introduction to advance care planning in practice. BMJ: Br Med J. 2013;347:f6064. 10.1136/bmj.f6064. 24144870 10.1136/bmj.f6064

[52] Schildmann J, Nadolny S, Haltaufderheide J, Gysels M, Vollmann J, Bausewein C. Ethical case interventions for adult patients. Cochrane Database Syst Rev. 2019;7(7):Cd012636. 10.1002/14651858.CD012636.pub2. 31424106 10.1002/14651858.CD012636.pub2 PMC6698942

[53] Suen MHP, Chow AYM, Woo RKW, Yuen SK. What makes advance care planning discussion so difficult? A systematic review of the factors of advance care planning in healthcare settings. Palliat Support Care. 2024;1–14. 10.1017/s1478951524000464. 10.1017/S1478951524000464 38766704

[54] Rhee JJ, Zwar NA, Kemp LA. Advance care planning and interpersonal relationships: a two-way street. Fam Pract. 2013;30(2):219–26. 10.1093/fampra/cms063. 23028000 10.1093/fampra/cms063

[55] Tulsky JA, Steinhauser KE, LeBlanc TW, Bloom N, Lyna PR, Riley J, et al. Triadic agreement about advanced cancer treatment decisions: perceptions among patients, families, and oncologists. Patient Educ Couns. 2022;105(4):982–6. 10.1016/j.pec.2021.08.001. 34384640 10.1016/j.pec.2021.08.001

[56] Wendler D, Rid A. Systematic review: the effect on surrogates of making treatment decisions for others. Ann Intern Med. 2011;154(5):336–46. 10.7326/0003-4819-154-5-201103010-00008. 21357911 10.7326/0003-4819-154-5-201103010-00008

[57] Bernacki RE, Block SD. Communication about serious illness care goals: A review and synthesis of best practices. JAMA Intern Med. 2014;174(12):1994–2003. 10.1001/jamainternmed.2014.5271. 25330167 10.1001/jamainternmed.2014.5271

[58] Moore E, Munoz-Arroyo R, Schofield L, Radley A, Clark D, Isles C. Death within 1 year among emergency medical admissions to Scottish hospitals: incident cohort study. BMJ Open. 2018;8(6):e021432. 10.1136/bmjopen-2017-021432. 29961029 10.1136/bmjopen-2017-021432 PMC6042622

[59] Bielinska AM, Archer S, Soosaipillai G, Riley J, Darzi LA, Urch C. Views of advance care planning in caregivers of older hospitalised patients following an emergency admission: A qualitative study. J Health Psychol. 2022;1359105320926547. 10.1177/1359105320926547. 10.1177/1359105320926547 PMC8793289 32515241

[60] Sævareid TJL, Førde R, Thoresen L, Lillemoen L, Pedersen R. Significance of advance care planning in nursing homes: views from patients with cognitive impairment, their next of kin, health personnel, and managers. Clin Interv Aging. 2019;14:997–1005. 10.2147/CIA.S203298. 31213786 10.2147/CIA.S203298 PMC6549780

