# Supplementary material for: Raising awareness and preparation for what may come: next of kin experiences of advance care planning with frail, home-dwelling older adults in geriatric units
Source: BMC Health Serv Res. 2025 Mar 27;25:454. doi: 10.1186/s12913-025-12609-9 (PMC11951624; doi:10.1186/s12913-025-12609-9)
Supplement: Supplementary file 2 — Additional file 2. Interview guide. [file 12913_2025_12609_MOESM2_ESM.docx]

**Interview guide for next of kin - Individual interview**

**Information and involvement**

- - Can you tell me about the advance care planning (ACP) you participated in (who was involved, where it took place, and what it was about)?
  - How were you invited to ACP? What were your thoughts about receiving that offer?
  - What do you think is the purpose of such a conversation? (Did you know what the conversation would be about? What do you believe it could mean for you?)

**Experiences and significance of the advance care planning (Positive/Negative)**

- - What is your experience participating in ACP? What aspects were positive or negative?
  - What significance do you think such a conversation has/can have? (now, later; for the patient; for next of kin.)
  - What role do you think hope and worries, what is important/valuable to you, thoughts about the last phase of life should have in such a conversation?
  - What are your thoughts on the topics discussed during the conversation?

**Facilitators and challenges/dilemmas**

- - Was there anything difficult to discuss? (If so, how did you experience it? - examples from the conversation)
  - What do you think can be challenging or problematic with ACP?
  - Have healthcare personnel asked the patient or you about the type of treatment and care the patient wants or does not want to go forward?

**Role of next of kin**

- - Did you gain any understanding of the role of next of kin in such conversations? (What do you think the role of next of kin is here?)
  - Now that you were involved in ACP, did you feel that your thoughts and opinions would have any significance for future decisions?
  - How much effort was made to consider what you know is important to the patient in choosing what to do next?
  - How well-informed are you about important decisions likely to be made regarding healthcare for your *spouse/mother/father* in the future (new hospital admissions or life-prolonging treatment, home assistance/home nursing, questions about assisted living or nursing home placement)?
  - What do you believe is your role in healthcare decisions?
  - What role have you played so far? What role do you think you should have?
  - What kind of information have you received about your role as next of kin if the patient were to lose the ability to consent?
  - Do you know what the legislation says about the role of next of kin

**Summary**

- - What do you think was the most important aspect that emerged during the ACP you participated in at the hospital?
  - What is necessary for you, as next of kin, and healthcare personnel to have a good collaboration?

**Areas for improvement**

- - What are your thoughts on the timing, considering your loved one’s situation/illness, for having such a conversation?
  - What do you think about the need for ACP? (for you as next of kin, for the patient)
  - Do you have any suggestions for improving ACP?

**Follow-up**

- - Have you/your family been informed about the possibility of having a new conversation regarding what is important for your *spouse/mother/father* in terms of future wishes and treatment? (renew/change the wishes)
  - What are your thoughts on participating in similar conversations in the future?
  - Who do you think should initiate such a conversation?
  - Is there anything you haven’t had a chance to say that you would like to share with those who were present? Do you have any other thoughts about ACP?
